# Severe destructive arthritis in adult-onset Still’s disease

**DOI:** 10.1093/rap/rky052

**Published:** 2019-01-15

**Authors:** Karima Abbaci Daghor, Abdelkrim Berrah

**Affiliations:** Internal Medicine Department, Mohamed Lamine Debaghine Hospital, BAB EL OUED University-Hospital Centre, Algiers University 1 Benyoucef Benkhada, BAB EL OUED, Algeria

**Fig. 1:**
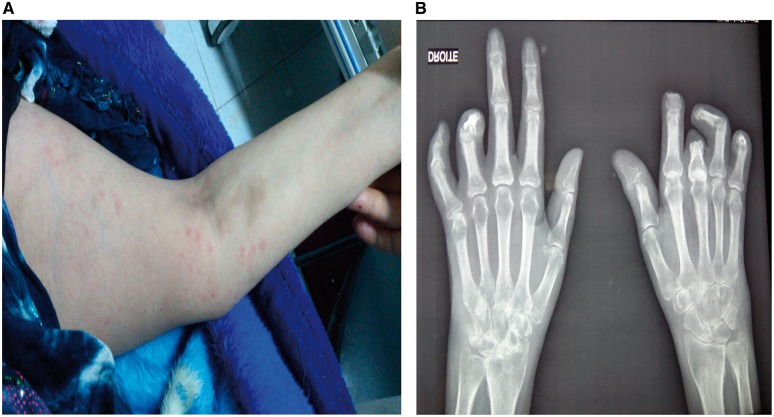
Destruction of the distal IP joints with ankylosis of the right wrist and evanescent rash. (**A**) Erythematous macules involving the right arm. (**B**) A radiograph of the hands shows severe structural destruction in the distal IP joints of both hands with erosions and ankylosis of the right wrist.

A 30 year old woman with a 2 year history of chronic adult-onset Still’s disease (AOSD) diagnosed according to Yamaguchi criteria (fever, polyarthritis, typical rash, sore throat and exclusion of infectious, autoimmune diseases or malignancy) had persistent symmetrical polyarthritis of the wrists, MCP, MTP and shoulder joints. She had no skin, scalp or nail psoriasis and there was no personal or family history of psoriasis, dactylitis or SpA. Also, the clinical profile was very different from a PsA: high fever, evanescent rash (Fig. 1A), pharyngitis and high serum ferritin with low glycosylated ferritin (1%). She had multiple flares of Still’s disease with high use of glucocorticoids and was dependent on these drugs. However, she had an allergy to MTX (high fever with skin rash). An X-ray of her hands (Fig. 1B) showed multiple subchondral erosions and narrowing of the joint spaces in the wrists with ankylosis in the right wrist and severe injuries with subluxations in the distal IP joints of the hands (first and fourth right fingers and first and fourth left fingers). We observed that the MCP joints were less injured. Articular sequelae appeared quickly (2 years) and caused poor functional outcomes. After poor response to tocilizumab, currently the patient is in remission with low-dose prednisone plus anakinra.

*Funding*: No specific funding was received from any funding bodies in the public, commercial or not-for-profit sectors to carry out the work described in this article.

*Disclosure statement*: The authors have declared no conflicts of interest.

